# Women’s preferences concerning IVF treatment: a discrete choice experiment with particular focus on embryo transfer policy

**DOI:** 10.1093/hropen/hoac030

**Published:** 2022-07-13

**Authors:** S Cornelisse, M S Vos, H Groenewoud, S Mastenbroek, L Ramos, D D M Braat, P F M Stalmeier, K Fleischer

**Affiliations:** Department of Obstetrics and Gynaecology, Radboud University Medical Centre, Nijmegen, The Netherlands; Department of Obstetrics and Gynaecology, Radboud University Medical Centre, Nijmegen, The Netherlands; Department of Health Evidence, Radboud University Medical Center, Nijmegen, The Netherlands; Department of Obstetrics and Gynaecology, Centre for Reproductive Medicine, Amsterdam Reproduction and Development Research Institute, Amsterdam UMC, University of Amsterdam, Amsterdam, The Netherlands; Department of Obstetrics and Gynaecology, Radboud University Medical Centre, Nijmegen, The Netherlands; Department of Obstetrics and Gynaecology, Radboud University Medical Centre, Nijmegen, The Netherlands; Department of Health Evidence, Radboud University Medical Center, Nijmegen, The Netherlands; Department of Obstetrics and Gynaecology, Radboud University Medical Centre, Nijmegen, The Netherlands

**Keywords:** IVF-ICSI outcome, embryo transfer, counseling, reproductive decision-making, assisted reproduction

## Abstract

**STUDY QUESTION:**

What outcomes are important for women to decide on the day of embryo transfer (ET) in IVF?

**SUMMARY ANSWER:**

The highest cumulative live birth rate (cLBR) per treatment was the most important treatment outcome for women undergoing an IVF treatment, regardless of the number of transfers needed until pregnancy and impact on quality of life.

**WHAT IS KNOWN ALREADY:**

Cleavage stage (Day 3) and blastocyst stage (Day 5) ETs are common transfer policies in IVF. The choice for one or the other day of ET differs between clinics. From the literature, it remains unclear whether the day of transfer impacts the cLBR. Patient preferences for the day of ET have not been examined yet.

**STUDY DESIGN, SIZE, AND DURATION:**

A discrete choice experiment (DCE) was performed to investigate female patients’ preferences and their values concerning various aspects of an IVF treatment, with a particular focus on ET policy. A multicenter DCE was conducted between May 2020 and June 2020 in which participants were asked to choose between different treatments. Each treatment was presented using hypothetical scenarios containing the following attributes: the probability of a healthy live birth per IVF treatment cycle, the number of embryos available for transfer (for fresh and frozen-thawed ET), the number of ETs until pregnancy and the impact of the treatment on the quality of life.

**PARTICIPANTS/MATERIALS, SETTING, METHODS:**

Women (n = 445) were asked to participate in the DCE at the start of an IVF treatment cycle in 10 Dutch fertility clinics. Participating women received an online questionnaire. The attributes’ relative importance was analyzed using logistic regression analyses.

**MAIN RESULTS AND THE ROLE OF CHANCE:**

A total of 164 women participated. The most important attribute chosen was the cLBR. The total number of embryos suitable for transfer also influenced women’s treatment preferences. Neither the number of transfers needed until pregnancy, nor the impact on quality of life influenced the treatment preferences in the aggregated data. For women in the older age group (age ≥36 years) and the multipara subgroup, the impact on quality of life was more relevant. Naive patients (patients with no prior experience with IVF treatment) assigned less value to the number of ETs needed until pregnancy and assigned more value to the cLBR than the patients who had experienced IVF.

**LIMITATIONS REASONS FOR CAUTION:**

An important limitation of a DCE study is that not all attributes can be included, which might be relevant for making choices. Patients might make other choices in real life as the DCE scenarios presented here are hypothetical and might not exactly represent their personal situation. We tried to avoid potential bias by selecting the attributes that mattered most to the patients obtained through patient focus groups. The final selection of attributes and the assigned levels were established using the input of an expert panel of professionals and by performing a pilot study to test the validity of our questionnaire. Furthermore, because we only included women in our study, we cannot draw any conclusions on preferences for partners.

**WIDER IMPLICATIONS OF THE FINDINGS:**

The results of this study may help fertility patients, clinicians, researchers and policymakers to prioritize the most important attributes in the choice for the day of ET. The present study shows that cLBR per IVF treatment is the most important outcome for women. However, currently, there is insufficient information in the literature to conclude which day of transfer is more effective regarding the cLBR. Randomized controlled trials on the subject of Day 3 versus Day 5 ETs and cLBR are needed to allow evidence-based counseling.

**STUDY FUNDING/COMPETING INTEREST(S):**

This work received no specific funding and there are no conflicts of interest.

**TRIAL REGISTRATION NUMBER:**

N/A.

WHAT DOES THIS MEAN FOR PATIENTS? Women going for IVF treatment might be confronted with the choice of day of embryo transfer (ET). In IVF, when deciding between an ET on Day 3 after oocyte retrieval (at cleavage stage of embryo development) or Day 5 after oocyte retrieval (at the blastocyst stage of embryo development), patients weigh multiple variables, such as the chance of pregnancy, the time to pregnancy, the safety of the treatment, its burden in daily life (quality of life) and the costs involved. This study aims to understand women’s preferences and, in doing so, prioritize issues that matter for patients.

Women starting IVF treatment were invited to take part in a survey and presented with a range of treatment scenarios. The factors taken into consideration per treatment scenario included the overall probability of a healthy live birth after one IVF treatment cycle (representing the cumulative live birth rate (cLBR)), number of embryos available for either fresh or frozen transfer, number of ETs needed until pregnancy (representing time to pregnancy) and the impact of the IVF treatment on their quality of life.

This study showed that patients highly value effectiveness in terms of the cLBR and the number of opportunities (number of embryos available for transfer), regardless of the treatment burden and time to pregnancy.

Currently, there is insufficient evidence in the literature to conclude which day of transfer is more effective regarding the cLBR. Randomized controlled trials on the subject are needed to inform and counsel patients about their treatment options.

## Introduction

As many as one-in-six couples experience infertility. This is defined as the failure to conceive after 1 year of unprotected intercourse (Zegers-Hochschild *et al.*, 2017). However, fertility interventions may be initiated in <1 year based on medical, sexual and reproductive history, age, physical findings and diagnostic testing (Zegers-Hochschild *et al.*, 2017). IVF (with or without ICSI) has evolved as one of the interventions of choice to help these couples. One of the most important steps in the IVF treatment is embryo transfer (ET) into the uterine cavity and cryopreservation of the surplus good quality embryos. Embryos can be transferred either on Day 3 (cleavage stage) or on Day 5 (blastocyst stage) of development. Surplus embryos suitable for transfer can be frozen for future use (Farquhar *et al.*, 2019).

IVF success rates are traditionally reported in terms of live birth per fresh ET (Maheshwari *et al.*, 2015; Maheshwari *et al.*, 2016; Glujovsky *et al.*, 2016; Glujovsky and Farquhar, 2016). Nowadays, it is assumed that from the patients' perspective, the cumulative live birth rate (cLBR) is more important, since it better summarizes the chance of a live birth over an entire treatment period (Maheshwari *et al.*, 2015, Malchau *et al.*, 2017; Malizia *et al.*, 2009). However, couples that opt for fertility treatment take other aspects of the treatment into account as well. They weigh, for example, the efficacy of the treatment, the costs involved, the safety, the physical and psychological treatment burden and patient centeredness (van Empel *et al.*, 2011; Huppelschoten *et al.*, 2014; Weiss *et al.*, 2017).

Currently available evidence suggests that the LBR after fresh blastocyst stage transfer is higher than after fresh cleavage stage transfer (Glujovsky *et al.*, 2022). However, extended culture *in vitro* (i.e. up to Day 5 instead of Day 3) also lowers the number of embryos available for transfer or cryopreservation, as some embryos will arrest in their development after Day 3 (Glujovsky *et al.*, 2022). Therefore, it is possible that the higher LBR after fresh ET in a blastocyst stage strategy does not translate into a higher cLBR. This cLBR includes the outcome of the fresh and frozen-thawed transferred embryos from one IVF treatment cycle. There is currently insufficient evidence to conclude which transfer strategy, cleavage stage or blastocyst stage is more effective regarding the cLBR (Glujovsky *et al.*, 2022; Cameron et al, 2020).

Until now, no data have been published on patients' preferences regarding the day of ET (Glujovsky and Farquhar, 2016; Maheshwari *et al.*, 2016). Such a study is needed to establish which fertility outcomes patients value most. One way to quantify the relative importance of attributes of a healthcare intervention is the use of a discrete choice experiment (DCE). This has proven to be a useful method to evaluate patients’ preferences (de Bekker-Grob, 2009). In a DCE, participants are shown certain hypothetical scenarios and are asked to indicate their preferred scenario. The choices of the participants reveal the value participants place on different attributes. In this study, a DCE was performed to investigate the female patients’ preferences and their values concerning various aspects of an IVF treatment, with a particular focus on ET policy. The possible differences in preference between subgroups were also examined. The results of this study may help fertility patients, clinicians, researchers and policymakers to prioritize the most important attributes in the choice for the day of ET.

## Materials and methods

### Study design

The DCE design of this study was based on a report of the International Society of Pharmacoeconomics and Outcomes Research (ISPOR) for good research practices for a Conjoint Analysis Task Force. This is a widely used guideline for designing a DCE study (Reed Johnson *et al.*, 2013; Hauber *et al.*, 2016). The DCE was designed to measure the value of different attributes concerning ET policies.

### Questionnaire development

The first step in the questionnaire development was the selection of attributes or elements of care most relevant for patients in an IVF treatment, with a particular focus on the ET policy. The selection of relevant attributes was based on a focus group with eight patients, together with input from two fertility doctors and one gynecologist. The final set of four attributes was determined in an expert meeting, consisting of three gynecologists, two embryologists and five fertility doctors working in one of the participating clinics. The attributes were chosen to be independent and had two or three levels. Levels correspond to the outcomes of attributes. These levels have to be realistic and sufficiently distinctive. Levels were based on the literature, consultation of experts, and finally agreed upon in the expert meeting. The attributes chosen were: the probability of a healthy live birth; total number of embryos available for transfer (either fresh or frozen); features about impact of treatment policy on quality of life; and the number of ETs needed until pregnancy. These four attributes cover the areas of ‘burden’ and ‘effectiveness’. A summary of the attributes and their levels is shown in Table I.

**Table I:** Final set of attributes and corresponding levels per IVF treatment used in the different scenarios.

No	Attributes*	Levels**
1	Probability of healthy live birth (cumulative live birth rate)	35%
		30%
		25%
2	Impact of treatment on quality of life	Low
		Moderate
		High
3	Number of embryos transfers needed until pregnancy	1 embryo transfer, equals 1 month until pregnancy
		Multiple embryo transfers, equals multiple months until pregnancy
4	Number of available embryos for transfer (either fresh or frozen)	6
		2

* Attribute: element of relevance for patients.

** Level: corresponds to possible outcomes of attributes in the discrete choice experiment.

Choice sets were derived from Table I. The four attributes and their levels generated a total of 36 (3 × 3 × 2 × 2) possible scenarios, enabling 1260 (36 × 35) possible combinations. The design was based on a 36-array orthogonal main effect plan (Street and Burgess, 2007). Orthogonality guarantees an optimal balance of the levels and attributes with a minimal correlation (Louviere *et al.*, 2007). The Ngene design software was used to draw the most efficient design (version 1.1.1 Choicemetrics Pty Ltd, Sydney, NSW, Australia). Seventeen of the 36 scenarios generated were dominant and were deleted. Of the 19 scenarios left, two different questionnaire versions with a near-orthogonal design were created. Patients were asked to choose between, scenarios A and B. As a result, each respondent gave 11 or 12 responses, which is reasonable, since previous studies indicate that respondents can handle up to 17 choice sets (Sculpher *et al.*, 2004; Bech *et al.*, 2011). An example of a choice between treatments A and B is given in Table II.

**Table II:** Example of a hypothetical choice set with scenarios A and B.

Characteristics of the IVF treatment cycle	Treatment A	Treatment B
Probability of a healthy live birth	30%	25%
Impact of treatment on quality of life	High	Moderate
Number of embryos transfers needed until pregnancy Represents time to pregnancy	1 embryo transfer, equals 1 month	1 embryo transfer, equals 1 month
Number of available embryos for transfer	2	6
I would choose this treatment	□	□

Rationality of participants’ choices was tested by adding one choice between two treatments, in which one treatment was logically better than the other (dominance test). To test internal consistency, one identical choice set was added (consistency test) in each of the two questionnaires.

Additional questions about baseline characteristics of patients, such as education level, parity, duration of infertility, number of previous IVF treatments and reason for fertility problem, were included. The questionnaire ended with an open question enabling patients to explain their answers and add comments.

### Pilot study

We performed a pilot study to test the questionnaire for difficulty, comprehensibility and inconsistencies (Lancsar and Louviere, 2008). This was carried out with a group of 20 patients from the outpatient clinic of reproductive healthcare of the Radboud University Medical Centre. These questionnaires were conducted face to face, enabling participants to ask questions and state difficulties with the questionnaire. Using this feedback we adapted the questions accordingly.

Basic analysis of the pilot data confirmed our expected direction of effect for all attributes. For the attribute ‘probability on a healthy live birth per IVF treatment’, a larger effect was seen than expected with much heterogeneity in response. We interpreted this as being caused by the relatively large differences in initially chosen levels: a 20% versus 30% versus 40% probability of conceiving with a healthy live birth. Therefore, the levels were adjusted to clinically more realistic ranges by narrowing the thresholds of the levels to 25% versus 30% versus 35% probability of a healthy live birth.

### Sample size calculation

We calculated the sample size by using a rule of thumb proposed by Lancsar and Louviere of 20 women per attribute (Lancsar and Louviere, 2008). Since our DCE contained four attributes, a minimum of 80 women was expected to be able to assess heterogeneity across choices.

### Study population

For our study, the patient population consisted of women referred for IVF treatment in one of the 10 participating Dutch clinics. In the Netherlands, government-funded treatments are the standard, and couples with idiopathic infertility and females aged below 38 years will first follow an expectant management and/or start with IUI with ovarian stimulation before there is an indication for IVF. All women starting IVF treatment were invited between May 2020 and June 2020 to participate in the study independent of the number of treatment cycle they started. Women had to be able to understand the Dutch questionnaire, as judged by the physician. If a respondent answered the illogical choice at the dominance test or showed inconsistency in the consistency test, this participant was excluded from analysis.

We invited only women of couples to participate, for logistic reasons. Owing to patient protection privacy rules, there was restricted access to the email address of partners. Therefore, conclusions on important outcomes and preferences can only be drawn for women in fertility care.

### Data collection

Women received an invitation by email to complete a digital questionnaire including an informed consent form. Data were anonymized using an online computer program (CASTOR EDC) that uses anonymous tokens so that no identifiable information is collected. If the questionnaire had not been returned within 2 weeks, a reminder was sent by email.

### Data analyses

Participants' preferences regarding IVF treatment and ET policy were analyzed using generalized estimating equations, an optimal method in cases of correlated responses (i.e. multiple choices per individual) (Burton *et al.*, 1998). Logistic regression analysis was performed to calculate coefficients for all four attributes, each coefficient representing the change in benefit of a one-level change in the attribute (Louviere *et al.*, 2007). First, we constructed a base model estimating the coefficients of the levels of all four attributes to determine whether a respondent considered an attribute as important. Then, we included interaction terms in this model to account for differences between subgroups of the respondent, consisting of age, treatment status and parity. Significance for multivariate analyses was set at *P* < 0.05. Analyses were performed using statistical analyses by SAS software (version 9.4, SAS Institute Inc., Cary, NC, USA) for Windows.

### Ethical approval

The institutional ethics committee of Radboud University Nijmegen Medical Centre provided ethical approval for this study (CMO nr 2020-6635).

## Results

A total of 445 women were invited to participate in the study; 194 women responded and completed the questionnaires, resulting in a response rate of 44%. The dominance test was answered illogically by six women. The consistency test was answered inconsistently by 21 women. Three patients failed in both tests. Therefore, 30 women were excluded from analysis, leaving 164 questionnaires for analyses.

The background characteristics of the women are presented in Table III. The mean age was 34 years (range 25–42 years). The majority of women was highly educated (75%) and were primary subfertile (62.8%). There were 55 women (33.5%) without prior IVF treatment and were therefore treatment-naive.

**Table III:** Baseline characteristics of female patients included in the discrete choice experiment.

Characteristic	Patients (N = 164)
Age (years)	34 (25-42)
Level of education(%)^a^:	
High	75
IVF Experience (%)^b^:	
Naive	33.5
Children (%):	
No	62.8
Cause of reduced fertility (%)^c^:	
Unexplained	32.3
Reduced sperm quality	36.6
Menstrual cycle disorder	4.3
Combined	7.3
Other	19.5

a High: higher professional education or university. Low and middle: no education, lower secondary education, secondary or intermediate vocational education.

b Naive, never had IVF treatment in the past.

^c^ If the patient had chosen two options in the questionnaire, in the combination ‘Reduced sperm quality’ with ‘Menstrual cycle disorder’ or ‘Other’, this was classified as ‘Combined’. Blocked tubes are presented in the category ‘Other’.

Table IV shows the results from the generalized estimating equations, using logistic regression analysis for the base model. The two attributes focusing on effectiveness had a significant impact on patients’ preferences for an ET policy; the cLBR (35% versus 25%, coefficient −2.27 [95% CI −2.01 to −2.52]) and number of embryos available for transfer (6 versus 2, coefficient −0.64 [95% CI −0.46 to −0.83]). The negative signs of those attributes indicate that patients were less likely to choose a treatment with lower pregnancy rates or fewer embryos available for transfer.

**Table IV:** Generalized estimating equations using logistic regression analysis for womens’ value per attribute.

Attributes	Level	Base model Coefficient (95% CI)	Exponentiated estimated Odd ratios (95% CI)	Risk ratio CI (5%)	Least squares means (95% CI)
Probability of healthy live birth (cumulative live birth rate)	35% vs.	Ref			76.0% (72.0–79.6%)
	30%	−0.79 (−0.57 to −1.01)*	2.21 (1.77 − 2.76)*	1.27	59.9% (56.0–61.8%)
	25%	−2.27 (−2.01 to −2.52)*	9.67 (7.47–12.5)*	3.07	24.7% (22.2–27.4%)
Impact of treatment on quality of life	Low vs.	Ref			54.1% (49.0–59.1%)
	Moderate	−0.003 (−0.24 to 0.24)	0.99 (0.75–1.27)	1.04	51.8% (49.0–54.7%)
	High	−0.09 (−0.32 to 0.14)	0.91 (0.75–1.15)	1.00	54.0% (50.2–57.7%)
Number of embryos transfers needed until pregnancy Represents time to pregnancy	One ET, equals one month vs.	Ref			54.8% (52.3–57.2%)
	Multiple ETs, equals multiple months	−0.12 (−0.31 to 0.08)	0.89 (0.73–1.08)	1.06	51.8% (47.7–56.0%)
Number of available embryos for transfer	6 vs. 2	Ref −0.64 (−0.46 to −0.83)*	1.90 (1.58–2.28)*	1.35	61.2% (57.3–64.8%) 45.3% (42.7–47.9%)

Coefficients were calculated using logistic regression analysis.

* *P* < 0.05.

Non-significant attributes were found for the attributes focusing on burden; the impact of the treatment on quality of life (low versus high, coefficient −0.09 [95% CI −0.32 to 0.14)] and the number of embryo transfers needed until pregnancy, representing time to pregnancy (one ET, equals 1 month versus multiple ETs, equals multiple months, coefficient −0.12 [95% CI −0.31 to 0.08]).

**Figure 1.**
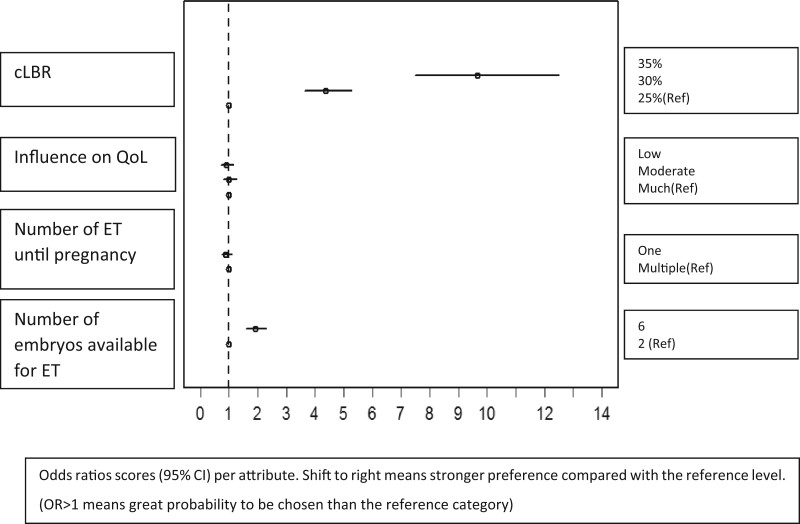
**Exponentiated estimated odds ratios per attribute.** cLBR, cumulative live birth rate; ET, embryo transfer; OR, odds ratio; QoL, quality of life; Ref, reference level.

Exponentiated estimated odds ratios (OR) show that, for example, the likelihood a woman chooses the scenario with a ‘cLBR of 35%’ is 9.67 times higher than the ‘cLBR of 25%’. Expressed as a risk ratio, the risk is 3.07 times higher that a patient chooses the highest probability rate of 35%, compared to the rate of 25%. This is also visible in the least square means: if in the scenario the probability of a live birth is 35%, the likelihood a patient chooses this scenario is 76.0% (Table IV). Exponentiated estimated ORs are plotted in Fig. 1.

Table V shows the results from the generalized estimating equations, using logistic regression analysis for the interaction model. There was a tendency in the age group of 36 years and above to place greater value on the impact of the quality of life attribute (OR 1.80 versus 0.88, *P* = 0.014). Treatment-naive patients assigned relatively less value to the number of ETs needed until pregnancy (OR 0.62 versus 1.06, *P* = 0.014) and assigned more value to the cLBR (OR 10.21 versus 9.64, *P* = 0.013). Multipara assigned more value to the cLBR (OR 20.6 versus 6.64, *P* < 0,001) and impact on the quality of life (OR 1.01 versus 0.87, *P* = 0.0033).

**Table V:** Patients value per attribute and subgroup: interaction model.

Attributes	Level	Exponentiated estimate odds ratios Age (years)			Exponentiated estimate odds ratios Naive			Exponentiated estimate odds ratios Parity		
		*<36*	*≥36*	*P*-Value	Naive	Non-naive	*P*-Value	Nullipara	Multipara	*P*-Value
Probability of a healthy live birth	35% versus 25%	8.57	13.18	0.32	10.21	9.64	0.013*	6.64	20.6	<0.001*
Impact of treatment on quality of life	Low versus high	0.88	1.80	0.014*	1.12	0.83	0.376	0.87	1.01	0.033*
Number of embryos transfers needed until pregnancy.	One embryo transfer, equals 1 month versus Multiple embryo transfers, equals multiple months	0.93	0.82	0.55	0.62	1.06	0.014*	0.93	0.79	0.435
Represents time to pregnancy										
Number of available embryos for transfer	6 versus 2	2.11	1.49	0.09	1.89	1.93	0.92	1.87	2.00	0.743

* *P* < 0.05.

## Discussion

To our knowledge, this is the first study that has used a DCE to quantify patients’ preferences in an IVF treatment in the context of the day of ET. Existing studies comparing the day of ET focused primarily on clinical outcomes, such as pregnancy, live birth rates and embryo freezing rate, with limited evidence in relation to the overall value patients’ place on alternative approaches to ET and their associated outcomes. Our study demonstrated that women place a high value on the cLBR per IVF treatment and the number of opportunities (number of embryos available for transfer), regardless of the treatment burden and time to pregnancy.

Our results are relevant for different stakeholders, i.e. infertile patients, fertility care professionals and policymakers, to focus more on the cLBR, especially since improved cryopreservation programmes increasingly contribute to IVF effectiveness (Wong *et al.*, 2014). Currently, in studies and national registry tables, the LBR per first fresh transfer is reported as most relevant primary outcome (Malizia *et al.*, 2009; Glujovsky and Farquhar, 2016; Maheshwari *et al.*, 2016). In contrast, comparative and reliable data on the cLBR are mostly unavailable.

The subgroup analyses revealed several differences between subgroups. For example, the impact of treatment on the quality of life attribute was relatively more important for the subgroups multiparity and the older age group. Furthermore, naive patients assigned relatively less value to the number of ETs needed until pregnancy and assigned more value to the cLBR; this latter difference may be explained by the fact that the naive group has no experience of the potential burden of the treatment and extra transfers as, for example, caused by the stressful waiting time between ET and pregnancy test or caused by failed implantation. When interpreting the subgroup analyses, it should be taken into account that the study focuses on patient preferences and not on demographic factors influencing the patient. Therefore, claims of causal directional influence between subgroups and the total population of infertile women cannot be made.

A strength of our study is the methodological DCE design, which was both orthogonal and balanced, with only a small correlation between attributes and with the informative value of an optimal design, following the checklist rapport of the ISPOR Conjoint Analysis Experimental Design Good Research Practices Task Force (Reed Johnson *et al.*, 2013). In addition, we included rationality and dominance tests. Treatment-naive and non-naive patients were included, since the patient preferences of both groups is of importance. One might argue that only patients who have experienced a certain disease or treatment are fully able to understand its burden and can make a balanced choice between the advantages and disadvantages of a particular treatment (Graziosi *et al.*, 2006). Others suggest that being a treatment-naive patient prevents any bias that women might have based their choices on, given their knowledge and experiences with previous treatments (Weiss *et al.*, 2017). We decided to include both groups in our study to give a realistic representation of the patient population, as most patients do need more than one IVF treatment for success. This gave the opportunity to study the possible influence on patient preferences of the experience of an unsuccessful earlier IVF treatment.

A limitation of our study is that the DCE scenarios will always be hypothetical for study participants and it is unclear whether they would make other choices in real life. To prevent this potential bias, we based our attributes and levels on both focus groups and the opinion of experts, which were adjusted after a pilot study. That said, other attributes may be important too, such as the chance of failure of an ET or the costs. We could not include the important attribute ‘the chance of failure of an embryo transfer’ since this attribute is confounded in a choice set with the attribute ‘the number of embryos available for transfer’ that would have led to unrealistic scenarios. We decided not to include the attribute ‘costs’ because, in the Dutch healthcare system, up to three IVF treatment cycles, inclusive of frozen-thawed ETs, are covered by basic health insurance. This should be taken into account in other countries with a different or with no reimbursement policy in IVF. If costs are an important factor for a patient, and the costs of frozen-thawed ETs are extra, the number of transfers needed until pregnancy hypothetically might become more important than the number of embryos available.

This DCE was performed during the coronavirus disease 2019 (COVID-19) pandemic, where IVF treatments were postponed or on hold owing to the restriction measures. It is known from recent literature that the COVID-19 pandemic has an impact on the emotional distress of fertility patients (Barra *et al.,* 2020). For that reason, we expected that the timing of the questionnaire could have an impact on the patients’ preferences, especially regarding their treatment burden. The treatment burden is expressed in the impact on the quality of life and, more specifically, to the number of ETs needed until pregnancy, which entails more negative pregnancy tests and a longer time to pregnancy. However, this study showed that for patients, even in times of elevated emotional distress, the cLBR is the most important factor, regardless of the treatment burden.

In this DCE study, only women were included, which is a limitation since, in most IVF treatments, a partner is involved who can influence the decision-making process. Previous research has shown that women and their partners have comparable preferences on quality of life and most aspects of care (Huppelschoten *et al.*, 2013; Holter *et al.*, 2014). At the same time, we cannot state that women and their partners will respond similarly on the DCE questionnaire and have the same preferences. Our results are therefore only applicable to women in fertility care and future research should focus on the potential effect of including their partners as well.

Another limitation is that the response rate was low to moderate, possibly owing to the timing of the questionnaire, during the COVID-19 pandemic. The low response rate could possibly lead to selection bias. On the other hand, the composition of our study population is comparable with other recently published DCEs (Braam *et al.,* 2020; van den Wijngaard *et al.*, 2015; Hentzen *et al.*, 2017; Weiss *et al.*, 2017).

### Implications for practice and future research

The study results might be helpful for the different stakeholders, i.e. fertility care professionals, researchers, subfertile patients and policymakers. Our results provide a clear picture of the preferences of the most important stakeholder in fertility care, the patient.

To enable patients to make an informed decision about their treatment with a specific focus on ET policy, information on all aspects of the treatment should be publicly available. Our study shows that women benefit from the following information: the cumulative number of live births per treatment, the average number of embryos (fresh and frozen-thawed) available for transfer and the time until pregnancy. Further studies are needed to clarify the cLBR, the costs and the patient burden associated with cleavage stage and with blastocyst stage transfer policy. We are currently conducting a multicenter randomized trial to provide more insight (Cornelisse *et al.*, 2021).

Nowadays, clinicians are increasingly aware of patients’ preferences and the necessity of shared decision-making. Our research can contribute to individual counseling of patients for a cleavage or blastocyst stage ET policy.

## Conclusion

Our study showed that women place a high value on the cLBR per IVF treatment and number of opportunities (number of embryos available for transfer), regardless of the treatment burden and time to pregnancy, in the context of the choice for day of ET. It is important to consider patient preferences when implementing any policy. Randomized controlled trials on ET policies are needed to allow evidence-based counseling and shared decision-making.

## Data Availability

The data underlying this article will be shared on reasonable request to the corresponding author.
